# A non-randomized pilot study protocol of a novel social support intervention for individuals in early recovery from hazardous alcohol use

**DOI:** 10.1371/journal.pone.0292293

**Published:** 2023-10-05

**Authors:** Li Yan McCurdy, Grace Kong, Suchitra Krishnan-Sarin, Brian D. Kiluk, Marc N. Potenza

**Affiliations:** 1 Department of Psychiatry, Yale School of Medicine, New Haven, Connecticut, United States of America; 2 Department of Radiology & Biomedical Imaging, Yale School of Medicine, New Haven, Connecticut, United States of America; 3 Yale Child Study Center, Yale School of Medicine, New Haven, Connecticut, United States of America; 4 Department of Neuroscience, Yale School of Medicine, New Haven, Connecticut, United States of America; 5 Wu Tsai Institute, Yale University, New Haven, Connecticut, United States of America; 6 Connecticut Mental Health Center, New Haven, Connecticut, United States of America; 7 Connecticut Council on Problem Gambling, Wethersfield, Connecticut, United States of America; PLoS ONE, UNITED STATES

## Abstract

**Introduction:**

Connecting individuals to recovery support services such as recovery community centers and mutual help organizations can be crucial for sustaining recovery from addiction. However, there may be barriers to engagement with recovery support services on individual (e.g., limited motivation) and structural (e.g., limited information on recovery resources) levels. This pilot study will determine the feasibility and acceptability of a novel online social support intervention (*Let’s Do Addiction Recovery Together*!, abbreviated as *LDART*) that uses pre-recorded videos created by members from several recovery support services to help individuals in early recovery from hazardous alcohol use sustain motivation during recovery and introduce them to freely available recovery support services in the community.

**Methods and analysis:**

This will be a non-randomized mixed-method pilot study. We will recruit 30 adults who engaged in past-year hazardous alcohol use and have some desire to cut down or quit to use LDART every night for a month. A subset of these participants will be invited to participate in a semi-structured qualitative interview after completing the study. Primary outcomes will be feasibility parameters such as recruitment and retention rates, and acceptability measures such as frequency of intervention use. Secondary outcomes will include self-reported changes in alcohol use, engagement in recovery support services, and quality of life at one-month post-intervention relative to baseline.

**Discussion:**

Results of this pilot study will inform a randomized controlled trial to examine efficacy of this intervention, with the goal of creating an accessible and scalable intervention that has direct benefits on individuals who want to cut down or quit problematic alcohol use.

**Trial registration:**

ClinicalTrials.gov ID: NCT06022107.

## Introduction

The impact of addiction on societies worldwide has been devastating and costly [1]. Existing pharmacological [2] and psychological [3, 4] interventions can be efficacious, but their efficacy is sometimes negated if deficits in social determinants of health such as social support are not concurrently addressed [5–7]. Social support is a core component of recovery capital, which refers to the internal and external resources needed for sustaining addiction recovery [8, 9]. Indeed, social isolation may worsen addiction outcomes, and social support typically statistically predicts addiction recovery [10–14]. Social support may be particularly important during the first few months of recovery, when relapse rates are high [15, 16].

One way of accruing social support is through engagement with recovery support services (RSSs), which are non-clinical, community-based, and often peer-led services. RSSs include mutual help organizations [17] (MHOs) which provide free support group meetings to help people in recovery from alcohol and other drugs. While 12-step programs such as Alcoholics Anonymous are arguably the most ubiquitous MHOs, others such as SMART Recovery and LifeRing have growing evidence supporting their effectiveness [18, 19]. Another category of RSSs is recovery community centers [20] that provide multiple services directly related to shorter-term recovery such as recovery coaching and support group meetings, as well as services that may sustain longer-term recovery by building recovery capital, such as employment assistance (e.g., guidance on resume writing, access to computers/internet, job listings) and recreational activities. This is consistent with a recent shift towards more holistic definitions of addiction recovery that encompass multiple domains of wellness beyond abstinence from drugs/alcohol [21]. Connecting individuals in early recovery to RSSs can be crucial for building recovery capital and sustaining addiction recovery [22–24].

Despite the importance, initiating and maintaining engagement with RSSs can be challenging for at least two reasons. First, there may be difficulties in finding and accessing RSSs due to individual factors (e.g., limited motivation, anxiety about entering environments full of strangers) and structural factors (e.g., limited information regarding recovery resources, limited understanding of whether services are free). Second, it can be difficult to stay motivated during the time-, emotion-, and effort-consuming process of finding and engaging in recovery-related resources, especially since this requires one to prioritize longer-term benefits of recovery over shorter-term benefits of substance use [25, 26]—tendencies often difficult for individuals with substance use disorders [27]. Such barriers may be overcome using a combination of community reinforcement approach [28] and contingency management [29], which equip individuals with hobbies and communities unrelated to alcohol use and provide individuals with immediate, salient rewards for reaching short-term recovery goals, respectively. While these are effective interventions [30], they have limited accessibility, as few treatment centers have the infrastructure or resources, few clinicians have the training to provide them, and they require individuals to be already engaged in formal treatment in order to gain access to these treatments [31].

The study team has developed a novel social support intervention that addresses these barriers to RSS engagement and maintaining recovery called *Let’s Do Addiction Recovery Together*! (*LDART*). LDART is a web-based intervention designed to assist people in early recovery by providing social reinforcement for reaching recovery goals and information about free recovery resources. LDART participants will be individuals who engage in hazardous alcohol use and want to cut down or quit. They will receive a short pre-recorded video every night for a month, contingent on them meeting their recovery goal for the day (e.g., not drinking, attending a support group meeting). These videos will congratulate and motivate the individual on their recovery journey in an immediately reinforcing way, similar to receiving monetary rewards for abstinence as in contingency management. Given that many in early recovery feel socially isolated [32], we hypothesize that these videos will be a potent social reward and motivator for reaching recovery goals. If the daily recovery goal is not met, they will receive an encouraging message instead. Irrespective of whether they reach their goal, they will also receive information about the RSS with which the creator of the video is associated, thereby introducing and “putting a face and a voice” to local RSSs and reducing informational and psychological barriers to engagement.

The aim of this study is to determine the feasibility and acceptability of LDART in supporting people in early recovery from hazardous alcohol use. Quantitative and qualitative measures will be collected to inform decisions to proceed with a RCT to assess efficacy of this intervention. The primary objectives of this study are to: i) determine feasibility in terms of recruitment, completing assessments, and study completion; and ii) determine acceptability of the intervention, in terms of whether it is used and well-received by participants. Secondary objectives of the study are to: iii) determine preliminary efficacy of this intervention on immediately relevant outcome variables such as past-month alcohol use and number of hours spent engaging with RSSs, and broader outcome variables such as quality of life and recovery capital; and iv) identify potential moderating factors (e.g., baseline levels of readiness to change, social support) that may influence preliminary efficacy.

## Materials and methods

### Study design

This protocol is written in accordance with the Standard Protocol Items: Recommendations for Interventional Trials (SPIRIT) reporting template [33] (S1 File). This pilot and feasibility study will be non-randomized, and all participants will receive the intervention (i.e., access the LDART website) for a few minutes every night for one month. This study will occur entirely remotely, using a combination of the Zoom platform for study visits and Qualtrics links for intervention delivery and questionnaire data collection. Post-intervention qualitative interviews (QIs) will occur on Zoom. This study has a mixed-methods design, including quantitative and qualitative evaluations of feasibility, acceptability and preliminary efficacy.

### Participants

#### Eligibility criteria

Participants will be 30 adults from the Greater New Haven area in Connecticut, USA. Past-year hazardous alcohol use will be defined as an Alcohol Use Disorders Identification Test (AUDIT [34]) score greater or equal to 8. Additionally, participants must have had at least one heavy drinking day (≥4 drinks for women; ≥5 drinks for men) in the past month, have some desire to cut down or quit drinking (>21 on the Recognition subscale of SOCRATES instrument), and have a device with access to the internet that they can reliably use every night for a month (e.g., a smartphone or computer).

Individuals with vulnerable population status (e.g., pregnant people, prisoners), or at the time of study participation are receiving in-patient psychiatric treatment involving hospitalization, or have experienced multiple instances of alcohol withdrawal in the past will be excluded from participation as they may require higher clinical care. Participants undergoing formal alcohol treatment at the time of study commencement will be excluded, but participants engaging with some community-based RSS will be included.

#### Sample size

We aim to recruit a sample size of 30 participants to use the intervention. Of them, 10 will be randomly selected for a follow-up QI. As this is a pilot study, a formal sample size calculation was not performed. Instead, sample size was inferred from other pilot studies of behavioral interventions [35]. Sample size for QIs was also derived from prior qualitative work.

#### Recruitment & consent

Participants will be recruited using flyers in the community, particularly at liquor stores, convenience stores, and other community spaces in Connecticut, USA. Potential participants will access a brief screening document on Qualtrics to determine eligibility. If eligible, they will schedule their first visit, where the study team will guide them through the consent process. If the potential participants agree, they will provide written informed consent to participate, documented in REDCap. They will receive an electronic copy of the consent form for their records. For QIs, participants will meet with a member of the study team on Zoom. They will review the information sheet and verbally consent to participating in the interview. They will receive an electronic copy of the information sheet for their records. The consent form and information sheet are provided in S1 File. Recruitment is anticipated to begin September 2023 until September 2024.

### Participant timeline

Timeline of each component of the study is depicted in Table 1. Briefly, the first visit on Zoom will entail participants meeting with a member of the study team to complete consent, baseline questionnaires, and assessment questionnaires on Qualtrics. They will then engage with the intervention for 28 days. Qualtrics links to assessment questionnaires will be emailed to them at post-intervention and one month post-intervention. After completing this study, interested participants will be invited to participate in a QI.

**Table 1:** Participant timeline.

	Pre-screening	Zoom Visit 1	Intervention	Post-intervention	1 month post-intervention	Optional Zoom Visit 2
Qualtrics: determine eligibility	**✓**					
• AUDIT						
• other inclusion/exclusion criteria						
Informed Consent		**✓**				
Baseline questionnaires		**✓**				
• Demographics						
• AUDIT						
• DAST						
• SOCRATES						
• MSPSS						
• MHO attendance history						
Intervention			**✓**			
Assessment questionnaires		**✓**		**✓**	**✓**	
• Timeline Follow Back: alcohol use						
• Timeline Follow Back: recovery services						
• WHOQOL-BREF						
• Assessment of Recovery Capital						
Acceptability assessments				**✓**		
Qualitative interview (optional)						**✓**

#### Participant compensation

Participants will be compensated $30 for completing Zoom Visit 1, which should take no more than one hour. Participants will be compensated $2 for logging onto LDART each night, or $15 for logging on all 7 nights on weeks 1 and 3 of the 4-week intervention, for a maximum of $30. They will complete questionnaires on Qualtrics on the last day of the intervention and one-month post-intervention for $30 each, which should each take less than half an hour to complete. They will receive an additional $20 if they participate in the QI. All compensation will be in the form of gift cards.

### Intervention

LDART is an intervention to be developed by the study team through informal feedback from academic and grassroots organizations. It will be a web-based social support intervention that can be accessed by smartphone or computer. It will be designed to support people in recovery from addiction by: 1) providing daily motivation for reaching their recovery goals, and 2) introducing them to freely available recovery resources in their community. The intervention will be thus designed to support individuals in recovery in the shorter term by providing daily rewards contingent on reaching recovery goals, and in the longer term by putting a face and voice to several RSSs, thereby reducing barriers to RSS engagement and increasing recovery capital.

The content for this intervention will be generated entirely by individuals from organizations that provide RSSs, who will be referred to this study as “support-providers”. They will be volunteers, employees and allies of these organizations such as recovery community centers such as Connecticut Community for Addiction Recovery (CCAR) and MHOs such as SMART Recovery and Recovery Dharma. They will be recruited by email and word of mouth and invited to pre-record a celebratory video for when the LDART participant meets their recovery goal for the day, and an encouraging video for days when their goal is not met. These videos will be generic, meaning that no protected health information from participants will be shared with support-providers. They will also be asked to provide information on the specific RSSs in which they are involved; for example, details on how to attend the support group meeting they facilitate, or how to access the recovery coaching service they provide at a recovery community center.

Screengrabs of the user interface of the intervention are provided in S1 File. Each night for a month, LDART participants will log onto the Qualtrics link and select a recovery goal for the next day (e.g., stay sober, drink less than usual, go to a support group meeting). The next night, they will return to the Qualtrics link to indicate whether they met their recovery goal for the day. If they answer yes, a celebratory video from a support-provider will be shown; if no, an encouraging message will be shown. Each video will be less than a minute long. The message may either be presented in video format or as a written transcript of the recorded video; this will vary from day to day to create variability and novelty. Participants will rate how motivating and supportive they found the message to be. Information provided by the support-provider about the details of their RSS will also be presented. Participants will then select their recovery goal for the next day. This entire process of using LDART should take less than 5 minutes each night. At the end of each week, a visual summary of how many goals the participant achieves (i.e., number of “bullseyes”) will be displayed so participants can track their progress.

### Measures

#### Baseline measures

The following data will be collected during the first study visit on zoom via questionnaires. Demographics: participants will report their age, gender, race, and ethnicity. The AUDIT [34], a 10-item questionnaire, will assess alcohol consumption and potential problems. A score of 8 or more is considered to indicate hazardous and harmful alcohol use and is used as an inclusion criteria for this study. The Drug Abuse Screening Test (DAST [36]), a 10-item instrument, quantifies negative consequences related to non-alcohol drug use. The Stages of Change Readiness and Treatment Eagerness Scale (SOCRATES [37]), a 19-item questionnaire, assesses readiness to change alcohol use. It has three subscales: recognition, ambivalence, and taking steps. The Multidimensional Scale of Perceived Social Support (MSPSS [38]), a 12-item measure, assesses perceived social support from family, friends, and significant others. MHO lifetime attendance history [39, 40] will be measured by showing a list of MHOs and RCCs in the Connecticut area and asking participants to indicate approximately how many meetings/activities they have attended at that organization and if they have heard of each organization.

#### Primary outcomes

Study feasibility will be assessed using the following data: a) time required to recruit the target number of participants, b) number of eligible participants required to recruit the required sample, c) rate of completion of the intervention, d) number of participants who complete follow-up assessments. To better understand barriers to participation, individual QIs will be conducted irrespective of whether the participant completes the study (as long as they consent to being contacted for the interview).

Acceptability of the intervention and study procedures will be assessed quantitatively and qualitatively. Quantitative data will be collected to investigate the number of times participants login to LDART during the month, particularly differences in login rates during weeks 1 and 3 (when log-ins are compensated) versus weeks 2 and 4 (when log-ins are not compensated) of the intervention. We will also collect preliminary measures of time spent on each page to gauge whether participants are engaging with the LDART content.

On the last day of the intervention, participants will complete an acceptability assessment questionnaire, adapted from a previously used questionnaire by the study team [41]. This comprises 9 questions (rated on a 5-point Likert scale) regarding the motivational and supportive properties of the video and message content, perceptions on the frequency of engaging with the intervention, and whether they enjoyed using the intervention. There will also be two open-ended questions for participants to indicate what they liked and did not like about the intervention and ways to improve it. Semi-structured individual QIs will also be utilized to determine participant perceptions of the intervention. To better understand barriers to intervention use, participants who withdraw from the study prior to completion will also be invited to take part in the qualitative study if they consent to being contacted.

#### Secondary outcomes

To assess preliminary efficacy, data will be collected via questionnaires at three timepoints: pre-intervention, post-intervention, and 1-month post-intervention. The Timeline Follow Back [42] (alcohol use) will provide specific dates for alcohol use in the past 28 days, and the number of standard drinks consumed on each drinking day, so as to have information on both frequency and quantity to calculate outcome variables such as percent days abstinent and percent heavy drinking days. The Timeline Follow Back (RSS engagement) will provide information on which days were spent engaging with RSSs in the past 28 days, for how many hours, and the type of RSS. The World Health Organization Quality of Life–Brief Version (WHOQOL-BREF [43]) is a 26-item questionnaire with four domains: physical health, psychological health, social relationships, and environment. The Brief Assessment of Recovery Capital [44] is a 10-item questionnaire measuring resources for initiating and sustaining recovery.

### Data collection, management, and monitoring

#### Data collection

Most data will be collected via standard and validated questionnaires sent to participants via Qualtrics. Participants will input their unique ID number when completing questionnaires, so all questionnaire data will be de-identified. QIs will be semi-structured individual interviews using a discussion guide, and recordings of the meeting will be collected using the embedded function in Zoom. Audio transcripts of the QIs will be de-identified (i.e., no names will appear in the transcript, only ID numbers).

#### Data management

The first author (LYM) will oversee data management. Only members on the study team who have all been trained in human subjects research will have access to the data. All data will be stored on devices compliant with the Health Insurance Portability and Accountability Act (HIPAA) and approved by the Yale Institutional Review Board (IRB), and data entry and management systems used will be secured and password protected. De-identified participant data will be transmitted to and stored on Yale-managed study computers. Participants’ identifying information (e.g., contact information) will be securely stored at each study site (in Qualtrics and Yale Secure Box) for internal use during the study. At the study conclusion, all records will continue to be securely located as dictated by the reviewing IRB, institutional policies, regulatory, or sponsor/funding agency requirements.

#### Data monitoring

The study principal investigators (PIs: LYM and MNP) are responsible for monitoring the data, ensuring protocol compliance, and conducting safety reviews. Data pertaining to recruitment, retention, follow-up rates, completeness, and availability of primary outcome data will be discussed at least monthly.

### Data analysis

Quantitative data will be imported into SPSS and/or Prism for analysis. Descriptive statistics will be used to describe demographic and categorical data, recruitment time, questionnaire completion rates and other outcome measures. Standard statistical analyses will be used to determine preliminary efficacy (e.g., random effects regression models) of continuously measured outcomes (e.g., percentage of heavy drinking days, quality of life), measured at three time points (prior to intervention, post-intervention, and one-month post-intervention). We will compare outcomes post-intervention versus pre-intervention; in a separate analysis, we will include outcomes at one-month post-intervention to evaluate the durability of the intervention effects.

Data from baseline questionnaires (e.g., perceived social support, lifetime MHO attendance) will be used in preliminary moderation analyses, to identify whether participants with certain baseline characteristics are associated with higher retention, acceptability, and preliminary efficacy.

Verbatim transcripts of the audio-recorded QIs will be analyzed via content analysis regarding study feasibility and acceptability. NVivo software will be used for coding and as a tool for data analysis. Two coders on the study team will develop code books independently, drawing from themes observed in the transcripts. The two coders will then merge their code books, resolving discrepancies by mutual agreement and with input from other researchers.

## Study status

This study will involve human participants, and ethical approval for this study was obtained from the Yale University Institutional Review Board (HIC#2000035434). A Certificate of Confidentiality was obtained through the National Institutes of Health. The trial is registered on ClinicalTrials.gov (NCT06022107). Recruitment and data collection have not yet begun and is planned to begin in September 2023.

## Discussion

This study will provide original data on a novel social support intervention designed to help people in early recovery from hazardous alcohol use. Quantitative and qualitative approaches will be used to comprehensively assess study outcomes. All major amendments to the protocol will be documented and submitted to Yale University Institutional Review Board for approval and documented in the trial registry at *ClinicalTrials*.*gov*. Research findings will be disseminated to the academic community through peer-reviewed journals and conference presentations. Results will also be disseminated to the addiction recovery community via RSSs and RSS conferences (e.g., Multiple Pathways of Recovery Conference) and via lay summaries on social media sites (e.g., the study Twitter page: @teamLDART). Data will be deposited to a public repository after study completion following publication of findings.

Adopting an implementation science approach early in the intervention development pipeline may increase the likelihood that interventions successfully transition from research to practice [45], to maximize the impact of the intervention. One consideration in developing LDART was for it to be web-based to maximize accessibility and scalability if results look promising. For example, its online and web-based platform can be accessed from multiple devices and does not require installing a mobile phone app. Likewise, the video uploading platform is designed so videos can be conveniently recorded and uploaded from a variety of devices, to reduce the steps needed to contribute videos. Since video content forms the foundation of the intervention, ensuring that sufficient videos are created will be important for temporal scalability (i.e., so that there will be more than a month’s worth of videos, so the intervention can be used for more than a month in the future).

Additional ways to reduce barriers to creating videos include having written consent waived, so that support-providers can create videos without having to schedule a time for the formal consenting process with a member of the study team. Instead, they will read the consenting document, complete a brief consent quiz, and then check a box indicating their consent. Finally, establishing and maintaining relationships with various organizations in the addiction recovery community is paramount for encouraging their involvement and contribution to this intervention, not only from the perspective of video creation, but also for the purposes of intervention development more generally. To that end, QIs will be held to get feedback on ways to encourage others to create videos, as well as feedback on the intervention. Their perspectives will be sought at various stages of development and optimization to maximize chances that the intervention is implementable in the community, as an additional way to reduce the research-to-practice gap that continues to persist in substance use disorder treatment development [46].

A limitation of this feasibility study is that limited conclusions may be drawn on the effectiveness of the intervention on outcomes. As an exploratory component to the study, measures of alcohol use and engagement in recovery support services will be collected before and after intervention use. Nonetheless, this study will provide important proof-of-concept data which will guide optimization of the intervention and decision-making regarding conducting an efficacy study.

## Supporting information

S1 File (PDF)
