# Supplementary material for: A non-randomized pilot study protocol of a novel social support intervention for individuals in early recovery from hazardous alcohol use
Source: PLoS One. 2023 Oct 5;18(10):e0292293. doi: 10.1371/journal.pone.0292293 (PMC10553253; doi:10.1371/journal.pone.0292293)
Supplement: S1 File — (PDF) [file pone.0292293.s001.pdf]

A non-randomized pilot study protocol of a novel social support intervention for individuals in early recovery from hazardous alcohol use

Li Yan McCurdy[1,2], Grace Kong[1], Suchitra Krishnan-Sarin[1], Brian D. Kiluk[1], Marc N. Potenza[1,3-7]

Supporting Information

- SPIRIT checklist (page 1-5)

- Screengrabs of LDART (page 6)

- Consent form for participating in the study (page 7-11)

- Consent form for participating in the QI (page 12-15)

SPIRIT 2013 Checklist: Recommended items to address in a clinical trial protocol and related documents*

Section/item	Item No	Description	Addressed on page number
**Administrative information**			
Title	1	Descriptive title identifying the study design, population, interventions, and, if applicable, trial acronym	1,3
Trial registration	2a	Trial identifier and registry name. If not yet registered, name of intended registry	3
	2b	All items from the World Health Organization Trial Registration Data Set	N/A
Protocol version	3	Date and version identifier	N/A
Funding	4	Sources and types of financial, material, and other support	2
Roles and responsibilities	5a	Names, affiliations, and roles of protocol contributors	1
	5b	Name and contact information for the trial sponsor	2
	5c	Role of study sponsor and funders, if any, in study design; collection, management, analysis, and interpretation of data; writing of the report; and the decision to submit the report for publication, including whether they will have ultimate authority over any of these activities	2
	5d	Composition, roles, and responsibilities of the coordinating centre, steering committee, endpoint adjudication committee, data management team, and other individuals or groups overseeing the trial, if applicable (see Item 21a for data monitoring committee)	N/A

Introduction

Background and rationale	6a	Description of research question and justification for undertaking the trial, including summary of relevant studies (published and unpublished) examining benefits and harms for each intervention	4-6
	6b	Explanation for choice of comparators	N/A
Objectives	7	Specific objectives or hypotheses	5-6
Trial design	8	Description of trial design including type of trial (eg, parallel group, crossover, factorial, single group), allocation ratio, and framework (eg, superiority, equivalence, noninferiority, exploratory)	7

Methods: Participants, interventions, and outcomes

Study setting	9	Description of study settings (eg, community clinic, academic hospital) and list of countries where data will be collected. Reference to where list of study sites can be obtained	8
Eligibility criteria	10	Inclusion and exclusion criteria for participants. If applicable, eligibility criteria for study centres and individuals who will perform the interventions (eg, surgeons, psychotherapists)	7
Interventions	11a	Interventions for each group with sufficient detail to allow replication, including how and when they will be administered	9-10
	11b	Criteria for discontinuing or modifying allocated interventions for a given trial participant (eg, drug dose change in response to harms, participant request, or improving/worsening disease)	N/A
	11c	Strategies to improve adherence to intervention protocols, and any procedures for monitoring adherence (eg, drug tablet return, laboratory tests)	N/A
	11d	Relevant concomitant care and interventions that are permitted or prohibited during the trial	7
Outcomes	12	Primary, secondary, and other outcomes, including the specific measurement variable (eg, systolic blood pressure), analysis metric (eg, change from baseline, final value, time to event), method of aggregation (eg, median, proportion), and time point for each outcome. Explanation of the clinical relevance of chosen efficacy and harm outcomes is strongly recommended	11-12
Participant timeline	13	Time schedule of enrolment, interventions (including any run-ins and washouts), assessments, and visits for participants. A schematic diagram is highly recommended (see Figure)	8-9

Sample size	14	Estimated number of participants needed to achieve study objectives and how it was determined, including clinical and statistical assumptions supporting any sample size calculations	8
Recruitment	15	Strategies for achieving adequate participant enrolment to reach target sample size	8

Methods: Assignment of interventions (for controlled trials)

Allocation:

Sequence generation	16a	Method of generating the allocation sequence (eg, computer-generated random numbers), and list of any factors for stratification. To reduce predictability of a random sequence, details of any planned restriction (eg, blocking) should be provided in a separate document that is unavailable to those who enrol participants or assign interventions	N/A
Allocation concealment mechanism	16b	Mechanism of implementing the allocation sequence (eg, central telephone; sequentially numbered, opaque, sealed envelopes), describing any steps to conceal the sequence until interventions are assigned	N/A
Implementation	16c	Who will generate the allocation sequence, who will enrol participants, and who will assign participants to interventions	N/A
Blinding (masking)	17a	Who will be blinded after assignment to interventions (eg, trial participants, care providers, outcome assessors, data analysts), and how	N/A
	17b	If blinded, circumstances under which unblinding is permissible, and procedure for revealing a participant's allocated intervention during the trial	N/A

Methods: Data collection, management, and analysis

Data collection methods	18a	Plans for assessment and collection of outcome, baseline, and other trial data, including any related processes to promote data quality (eg, duplicate measurements, training of assessors) and a description of study instruments (eg, questionnaires, laboratory tests) along with their reliability and validity, if known. Reference to where data collection forms can be found, if not in the protocol	12
	18b	Plans to promote participant retention and complete follow-up, including list of any outcome data to be collected for participants who discontinue or deviate from intervention protocols	9

Data management	19	Plans for data entry, coding, security, and storage, including any related processes to promote data quality (eg, double data entry; range checks for data values). Reference to where details of data management procedures can be found, if not in the protocol	12-13
Statistical methods	20a	Statistical methods for analysing primary and secondary outcomes. Reference to where other details of the statistical analysis plan can be found, if not in the protocol	13
	20b	Methods for any additional analyses (eg, subgroup and adjusted analyses)	N/A
	20c	Definition of analysis population relating to protocol non-adherence (eg, as randomised analysis), and any statistical methods to handle missing data (eg, multiple imputation)	N/A
**Methods: Monitoring**			
Data monitoring	21a	Composition of data monitoring committee (DMC); summary of its role and reporting structure; statement of whether it is independent from the sponsor and competing interests; and reference to where further details about its charter can be found, if not in the protocol. Alternatively, an explanation of why a DMC is not needed	13
	21b	Description of any interim analyses and stopping guidelines, including who will have access to these interim results and make the final decision to terminate the trial	N/A
Harms	22	Plans for collecting, assessing, reporting, and managing solicited and spontaneously reported adverse events and other unintended effects of trial interventions or trial conduct	N/A
Auditing	23	Frequency and procedures for auditing trial conduct, if any, and whether the process will be independent from investigators and the sponsor	N/A
**Ethics and dissemination**			
Research ethics approval	24	Plans for seeking research ethics committee/institutional review board (REC/IRB) approval	13-14
Protocol amendments	25	Plans for communicating important protocol modifications (eg, changes to eligibility criteria, outcomes, analyses) to relevant parties (eg, investigators, REC/IRBs, trial participants, trial registries, journals, regulators)	14

Consent or assent	26a	Who will obtain informed consent or assent from potential trial participants or authorised surrogates, and how (see Item 32)	8
	26b	Additional consent provisions for collection and use of participant data and biological specimens in ancillary studies, if applicable	N/A
Confidentiality	27	How personal information about potential and enrolled participants will be collected, shared, and maintained in order to protect confidentiality before, during, and after the trial	12-13
Declaration of interests	28	Financial and other competing interests for principal investigators for the overall trial and each study site	2
Access to data	29	Statement of who will have access to the final trial dataset, and disclosure of contractual agreements that limit such access for investigators	13
Ancillary and post-trial care	30	Provisions, if any, for ancillary and post-trial care, and for compensation to those who suffer harm from trial participation	N/A
Dissemination policy	31a	Plans for investigators and sponsor to communicate trial results to participants, healthcare professionals, the public, and other relevant groups (eg, via publication, reporting in results databases, or other data sharing arrangements), including any publication restrictions	14
	31b	Authorship eligibility guidelines and any intended use of professional writers	N/A
	31c	Plans, if any, for granting public access to the full protocol, participant-level dataset, and statistical code	N/A
**Appendices**			
Informed consent materials	32	Model consent form and other related documentation given to participants and authorised surrogates	SI
Biological specimens	33	Plans for collection, laboratory evaluation, and storage of biological specimens for genetic or molecular analysis in the current trial and for future use in ancillary studies, if applicable	N/A

*It is strongly recommended that this checklist be read in conjunction with the SPIRIT 2013 Explanation & Elaboration for important clarification on the items. Amendments to the protocol should be tracked and dated. The SPIRIT checklist is copyrighted by the SPIRIT Group under the Creative Commons "Attribution-NonCommercial-NoDerivs 3.0 Unported" license.

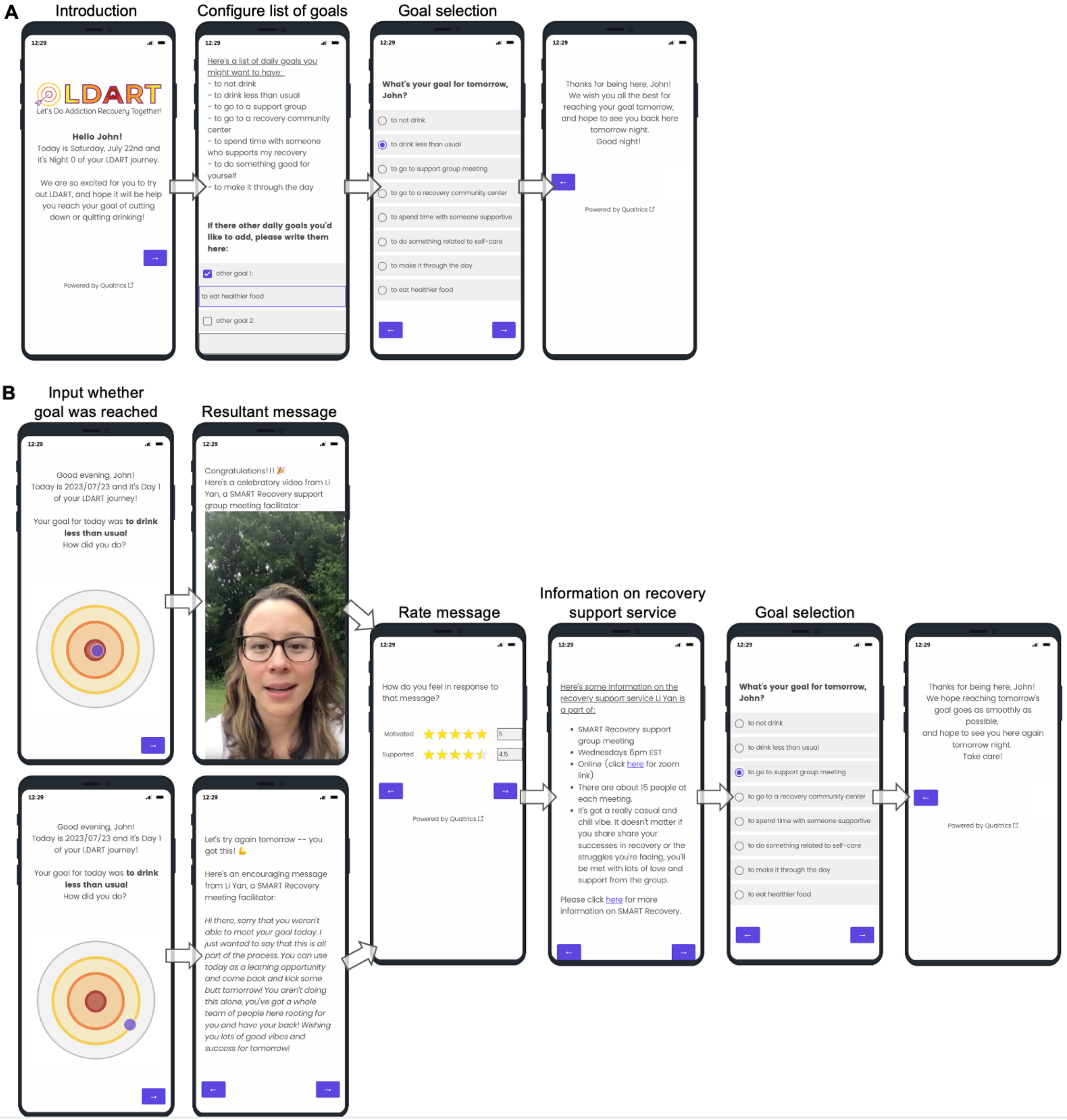

Figure 1. Screengrabs of LDART intervention. A) The night before beginning the intervention, participants will log into LDART to customize recovery goals and to set a recovery goal for the next day. **B)** The next night, they will log into LDART to report whether they met their recovery goal by clicking on a dartboard -- bullseye if they reached their goal, elsewhere if they did not. Depending on what they select, a celebratory video or an encouraging message will be displayed, respectively. Participants will then be asked to rate how motivating and supportive they found that message to be. Information on the support-provider's recovery support service will be displayed, and participants will be asked to select a goal for the next day.

COMPOUND AUTHORIZATION AND CONSENT FOR PARTICIPATION IN A RESEARCH STUDY

YALE UNIVERSITY

Study Title: Piloting a novel social support intervention for addiction recovery
Principal Investigator (the person who is responsible for this research): Li Yan McCurdy, PhD; 1 Church St, 7th floor, New Haven, CT 06510

Research Study Summary:

- We are asking you to join a research study.
- The purpose of this research study is to test whether using a web-based intervention is feasible, acceptable and helpful for people who engage in hazardous drinking trying to cut down or quit drinking.
- Study activities will include: using the intervention for a few minutes each night for one month, and filling in questionnaires about who you are, your drinking habits, and your support network.
- Your involvement will require 5.5 hours in total.
- There may be some risks from participating in this study. There may be breaches of your confidentiality, however this is unlikely. You may feel upset when answering questions about yourself, but you will be able to skip any questions you do not want to answer.
- The study may benefit you. As part of this study, you will receive encouragement and information that may help you cut down or quit drinking.
- Taking part in this study is your choice. You can choose to take part, or you can choose not to take part in this study. You also can change your mind at any time. Whatever choice you make will not have any effect on your relationship with Yale University.
- If you are interested in learning more about the study, please continue reading, or have someone read to you, the rest of this document. Ask the study staff questions about anything you do not understand. Once you understand the study, we will ask you if you wish to participate; if so, you will have to sign this form.

Why is this study being offered to me?
We are asking you to take part in a research study because you are an adult in Connecticut who engaged in hazardous drinking in the past year and have some desire to cut down or quit drinking. We are looking for 20 participants to be part of this research study.

Who is paying for the study?
This study is paid for by the Society of Addiction Psychology, which is a part of the American Psychological Association.

What is the study about?
The purpose of this study is to test whether using a web-based intervention is feasible, acceptable and helpful for people who engage in hazardous drinking trying to cut down or quit

drinking. The goal is to create a freely-available resource that provides daily motivation and information to help people in early recovery reach their recovery goals.

What are you asking me to do and how long will it take?
If you agree to take part, your participation in this study will involve 1) filling in questionnaires about who you are, your drinking habits, and your support network, 2) using the intervention for a few minutes each night for a month (which entails answering whether you reached your recovery goal for the day, and receiving information and encouragement to support your recovery), and 3) filling in the same questionnaires at the end of the intervention, and again one month later. We think that the study will take a total of 5.5 hours of your time.

Are there any risks from participating in this research?
If you decide to take part in this study, there are some risks. This intervention is focused on alcohol use, which some may find upsetting to think about. While unlikely, you may feel distress when using the intervention or filling in questionnaires. To prevent this, the intervention is carefully designed and monitored to ensure nothing distressing will occur, and you can skip any questions for any reason. There may be breaches of your confidentiality. However, this is unlikely as all data you will provide will not have your personal information attached to it. We do not expect any physical risks from taking part in this study.

How can the study possibly benefit me or others?
You may benefit from taking part in this study. When using the intervention, you will receive supportive and motivating videos and/or information about recovery support services each night for a month, which may keep you motivated in your recovery as well as decrease barriers to these recovery services. Taking part in an intervention study that may improve treatment outcomes for others with alcohol use issues may be considered a benefit. This study will also provide important evidence as to whether this intervention may be helpful for individuals who engage in hazardous alcohol use.

Are there any costs to participation?
You will not have to pay for taking part in this study. The only cost may be your time participating in this study.

Will I be paid for participation?
You will be paid for taking part in this study. You will receive a $30 gift card for filling in questionnaires at the start of the intervention. You will receive $2/night for clicking on the intervention link and completing the activities, or $15/week if you do it on all nights each week. (You will receive this as one lump sum at the end of the intervention.) You will receive a $25 gift card for completing questionnaires at the end of the intervention, and another $25 gift card for completing the same questionnaires one month later. In total, you may receive up to $140 in gift cards for participating in this study. According to the rules of the Internal Revenue Service (IRS), payments for taking part in a study may be considered taxable income.

How will you keep my data safe and private?

All of your responses will be held in confidence. Only the researchers involved in this study and those responsible for research oversight (such as representatives of the Yale University Human Research Protection Program, the Yale University Institutional Review Boards, and others) will have access to any information that could identify you that you provide. We will share it with others if you agree to it or when we have to do it because U.S. or State law requires it. For example, we will tell somebody if we learn that you are hurting a child or an older person.

Data will be stored on password-protected, Yale-managed computers. All questionnaire data and data about how often you used the intervention will be linked to your participant ID and not your identifying information like your name. Your identifying information will not be stored in the same file as your data.
When we publish the results of the research or talk about it in conferences, we will not use your name. If we want to use your name, we would ask you for your permission. We will also share information about you with other researchers for future research but we will not use your name or other identifiers. We will not ask you for any additional permission.

Identifiers might be removed from the identifiable private information, and after such removal, the information could be used for future research studies or distributed to another investigator for future research studies without additional informed consent from you.

What Information Will You Collect About Me in this Study?

The information we are asking to use and share is called "Protected Health Information." It is protected by a federal law called the Privacy Rule of the Health Insurance Portability and Accountability Act (HIPAA). In general, we cannot use or share your health information for research without your permission. If you want, we can give you more information about the Privacy Rule. Also, if you have any questions about the Privacy Rule and your rights, you can speak to Yale Privacy Officer at 203-432-5919.

The specific information about you and your health that we will collect, use, and share includes:

- Research study records
- Information obtained during this research regarding
    - How often you use the intervention
    - Information on your alcohol and other drug use and other behaviors when using the intervention and from filling in questionnaires.

How will you use and share my information?

We will use your information to conduct the study described in this consent form.
We may share your information with:

- The U.S. Department of Health and Human Services (DHHS) agencies
- Representatives from Yale University, the Yale Human Research Protection Program and the Institutional Review Board (the committee that reviews, approves, and monitors research on human participants), who are responsible for ensuring research compliance. These individuals are required to keep all information confidential.

- Principal Investigator of the study
- Co-Investigators and other investigators
- Study Coordinator and Members of the Research Team
- Data and Safety Monitoring Boards and others authorized to monitor the conduct of the Study

We will do our best to make sure your information stays private. But, if we share information with people who do not have to follow the Privacy Rule, your information will no longer be protected by the Privacy Rule. Let us know if you have questions about this. However, to better protect your health information, agreements are in place with these individuals and/or companies that require that they keep your information confidential.

Why must I sign this document?
By signing this form, you will allow researchers to use and disclose your information described above for this research study. This is to ensure that the information related to this research is available to all parties who may need it for research purposes. You always have the right to review and copy your health information in your medical record.

What if I change my mind?
The authorization to use and disclose your health information collected during your participation in this study will never expire. However, you may withdraw or take away your permission at any time. You may withdraw your permission by telling the study staff or by writing to Li Yan McCurdy at liyan.mccurdy@yale.edu or LDART@yale.edu

If you withdraw your permission, you will not be able to stay in this study but the care you get from your doctor outside this study will not change. No new health information identifying you will be gathered after the date you withdraw. Information that has already been collected may still be used and given to others until the end of the research study to ensure the integrity of the study and/or study oversight.

What if I want to refuse or end participation before the study is over?
Taking part in this study is your choice. You can choose to take part, or you can choose not to take part in this study. You also can change your mind at any time. Whatever choice you make will not have any effect on your relationship with Yale University.

Who should I contact if I have questions?
Please feel free to ask about anything you don't understand. If you have questions later or if you have a research-related problem, you can email the Principal Investigator at liyan.mccurdy@yale.edu

If you have questions about your rights as a research participant, or you have complaints about this research, you call the Yale Institutional Review Boards at (203) 785-4688 or email hrpp@yale.edu.

Authorization and Documentation of Consent

Your signature below indicates that you read and understand this consent form and the information presented and that you agree to be in this study.

We will email you a copy of this form. Participant email address: ________________

______________________	______________________	________
Participant Printed Name	Participant Signature	Date
______________________	______________________	________
Person Obtaining Consent Printed Name	Person Obtaining Consent Signature	Date

If you decide not to consent, would you be willing to tell us why?
- Time commitment: one month is too long
- Time commitment: every night is too frequent
- Don't like the idea of watching videos of people in my community
- Am concerned about confidentiality/privacy, that my data will be leaked
- Others: ___________

Irrespective of whether I consent to taking part in this study, I consent to being emailed by the study team about participating in a 30-minute interview about my experiences in this study, for which I would receive a $40 gift card. YES/NO

INFORMATION SHEET FOR PARTICIPATION IN A RESEARCH STUDY

YALE UNIVERSITY

Study Title: Piloting a novel social support intervention for addiction recovery
Principal Investigator (the person who is responsible for this research): Li Yan McCurdy, PhD; 1 Church St, 7th floor, New Haven, CT 06510

Research Study Summary:

- We are asking you to join a research study.

- The purpose of this research study is to collect feedback on the experiences associated with contributing to and using *Let's Do Addiction Recovery Together* (LDART), and to use this information to improve the intervention.

- Study activities will include: participating in an individual interview which will take place on Zoom (a secure online videoconferencing platform). If you were involved in the contribution of content to LDART, you will be asked questions about what the process was like, how to make it easier, and thoughts about the intervention name. If you used LDART, you will be asked questions about ways it was and was not useful to your recovery, ways to make it more helpful, and thoughts about the intervention name.

- We will audio and possibly video record the discussion. The audio tape will be transcribed and any identifying information (e.g., your name) will be left out of the transcription for your privacy and confidentiality. The audio and video tapes will be destroyed shortly after transcription.

- Your involvement will require 30 minutes.

- We do not expect any physical risks from taking part in this study. Some may experience emotional distress when talking about their experiences. To prevent this, you do not have to respond to any question that makes you uncomfortable or that you otherwise do not want to answer. There is also a possible risk of loss of confidentiality. To prevent this, transcripts of the interview will not contain your name, only your participant ID.

- The study may have no benefits to you. However, we hope that your participation will assist in improving LDART and will help individuals stay in recovery from hazardous alcohol use.

- Taking part in this study is your choice. You can choose to take part, or you can choose not to take part in this study. You also can change your mind at any time. Whatever choice you make will not have any effect on your relationship with Yale University.

- If you are interested in learning more about the study, please continue reading, or have someone read to you, the rest of this document. Ask the study staff questions about anything you do not understand. Once you understand the study, we will ask you if you wish to participate; if so, we will continue with the study procedures described in this form.

<u>Are there any costs to participation? Will I be paid for participation?</u>
You will not have to pay to take part in this study. The only costs may include transportation and your time coming to the study visits. You will be paid $40 in an electronic gift card (e.g., Amazon) after completing the interview, for taking part in the study.

<u>How will you keep my data safe and private?</u>
All of your responses will be held in confidence. Only the researchers involved in this study and those responsible for research oversight (such as representatives of the Yale University Human Research Protection Program, the Yale University Institutional Review Boards, and others) will have access to any information that could identify you that you provide. We will share it with others if you agree to it or when we have to do it because U.S. or State law requires it. For example, we will tell somebody if we learn that you are hurting a child or an older person.

All audio recordings and transcripts will be collected and stored on password-protected, Yale-managed computers, using HIPAA-compliant software (e.g., Zoom, Yale Box). These data can only be accessed by members of the research team directly involved in this project.

Data will be de-identified with unique codes and a key in a document separate from the data. Specifically, confidentiality of sensitive or health-related information collected via surveys will be accomplished by assigning unique numeric identifiers to each subject, and exclusively using these numbers on all electronic data records which contain sensitive information. For example, demographic data in Qualtrics will be labeled using participants' unique numeric identifier (assigned to them at the time of enrollment) to keep their identifying information and their survey responses separate. Transcripts of interviews will not include names of participants but rather their numeric identifiers.

When we publish the results of the research or talk about it in conferences, we will not use your name. If we want to use your name, we would ask you for your permission.

We will keep your information confidential. We ask that all focus group members not repeat any information shared during the focus group to others. However, we have no control over what happens outside of the group. Therefore, please, be aware of what you share in the group and do not share anything you hear from others outside of the group.

We may also share information about you with other researchers for future studies, but we will not use your name or any other identifiers. We will not ask you for any additional permissions.

<u>What if I want to refuse or end participation before the study is over?</u>

Taking part in this study is your choice. You can choose to take part, or you can choose not to take part in this study. You also can change your mind at any time. Whatever choice you make will not have any effect on your relationship with Yale University. You do not give up any of your legal rights by giving your verbal agreement to participate.

Who should I contact if I have questions?

Please feel free to ask about anything you don't understand.

If you have questions later or if you have a research-related problem, you can email the Principal Investigator at liyan.mccurdy@yale.edu.

If you have questions about your rights as a research participant, or you have complaints about this research, you call the Yale Institutional Review Boards at (203) 785-4688 or email hrpp@yale.edu.

What Information Will You Collect About Me in this Study?

The information we are asking to use and share is called "Protected Health Information." It is protected by a federal law called the Privacy Rule of the Health Insurance Portability and Accountability Act (HIPAA). In general, we cannot use or share your health information for research without your permission. If you want, we can give you more information about the Privacy Rule. Also, if you have any questions about the Privacy Rule and your rights, you can speak to Yale Privacy Officer at 203-432-5919.

The specific information about you and your health that we will collect, use, and share includes audio and video recordings of the interview.

How will you use and share my information?

We will use your information to conduct the study described in this consent form.

We may share your information with:

- The U.S. Department of Health and Human Services (DHHS) agencies
- Representatives from Yale University, the Yale Human Research Protection Program and the Institutional Review Board (the committee that reviews, approves, and monitors research on human participants), who are responsible for ensuring research compliance. These individuals are required to keep all information confidential.
- The Principal Investigator
- Co-Investigators and other investigators
- Study Coordinator and Members of the Research Team
- Data and Safety Monitoring Boards and others authorized to monitor the conduct of the Study

We will do our best to make sure your information stays private. But, if we share information with people who do not have to follow the Privacy Rule, your information will no longer be protected

by the Privacy Rule. Let us know if you have questions about this. However, to better protect your health information, agreements are in place with these individuals and/or companies that require that they keep your information confidential.

Why must I agree to the use of my information?

By giving permission for the use of your health information, you will allow researchers to use and disclose your information described above for this research study. This is to ensure that the information related to this research is available to all parties who may need it for research purposes. You always have the right to review and copy your health information in your medical record.

What if I change my mind?

The authorization to use and disclose your health information collected during your participation in this study will never expire. However, you may withdraw or take away your permission at any time. You may withdraw your permission by telling the study staff or by writing to Li Yan McCurdy at liyan.mccurdy@yale.edu or LDART@yale.edu.

If you withdraw your permission, you will not be able to stay in this study but the care you get from your doctor outside this study will not change. No new health information identifying you will be gathered after the date you withdraw. Information that has already been collected may still be used and given to others until the end of the research study to ensure the integrity of the study and/or study oversight.

DO WE HAVE YOUR CONSENT TO BEGIN THIS STUDY?
