# Supplementary material for: A non-randomized pilot study protocol of a novel social support intervention for individuals in early recovery from hazardous alcohol use
Source: PLoS One. 2023 Oct 5;18(10):e0292293. doi: 10.1371/journal.pone.0292293 (PMC10553253; doi:10.1371/journal.pone.0292293)
Supplement: Peer review history

# Editor report: Decision Letter 0

**DOI:** 10.1371/journal.pone.0292293.r001

**Authors:** Vanessa Carels

18 Sep 2023

A non-randomized pilot study protocol of a novel social support intervention for individuals in early recovery from hazardous alcohol use

PONE-D-23-29288

Dear Dr. McCurdy,

We’re pleased to inform you that your manuscript has been judged scientifically suitable for publication and will be formally accepted for publication once it meets all outstanding technical requirements.

Within one week, you’ll receive an e-mail detailing the required amendments. When these have been addressed, you’ll receive a formal acceptance letter and your manuscript will be scheduled for publication.

An invoice for payment will follow shortly after the formal acceptance. To ensure an efficient process, please log into Editorial Manager at http://www.editorialmanager.com/pone/, click the 'Update My Information' link at the top of the page, and double check that your user information is up-to-date. If you have any billing related questions, please contact our Author Billing department directly at authorbilling@plos.org.

If your institution or institutions have a press office, please notify them about your upcoming paper to help maximize its impact. If they’ll be preparing press materials, please inform our press team as soon as possible -- no later than 48 hours after receiving the formal acceptance. Your manuscript will remain under strict press embargo until 2 pm Eastern Time on the date of publication. For more information, please contact onepress@plos.org.

Kind regards,

Vanessa Carels

Staff Editor

PLOS ONE

Journal requirements:

1. Thank you for stating the following in the Competing Interests section: "I have read the journal's policy and the authors of this manuscript have the following competing interests: BDK has received personal fees from the Analgesic, Anesthetic, and Addiction Clinical Trial Translations, Innovations, Opportunities, and Networks (ACTTION) and from CBT4CBT and Sparian Biosciences outside the submitted work; and has received research support from the Mohegan Sun Casino. MNP has consulted for Opiant Therapeutics, Game Day Data, Baria-Tek, the Addiction Policy Forum, AXA and Idorsia Pharmaceuticals; been involved in a patent application with Yale University and Novartis; received research support from the Mohegan Sun Casino, Children and Screens and the Connecticut Council on Problem Gambling; and consulted for legal and gambling entities on issues related to impulse control, internet use and addictions. The other authors report no disclosures. All authors declare no competing interests with respect to the content of this manuscript".

Please confirm that this does not alter your adherence to all PLOS ONE policies on sharing data and materials, by including the following statement: This does not alter our adherence to  PLOS ONE policies on sharing data and materials.” (as detailed online in our guide for authors http://journals.plos.org/plosone/s/competing-interests). If there are restrictions on sharing of data and/or materials, please state these. Please note that we cannot proceed with consideration of your article until this information has been declared. 

Please respond by return email with your amended Competing Interests Statement and we will change the online submission form on your behalf.

# Editor report: Acceptance letter

**DOI:** 10.1371/journal.pone.0292293.r002

**Authors:** Vanessa Carels

26 Sep 2023

PONE-D-23-29288 

A non-randomized pilot study protocol of a novel social support intervention for individuals in early recovery from hazardous alcohol use 

Dear Dr. McCurdy:

I'm pleased to inform you that your manuscript has been deemed suitable for publication in PLOS ONE. Congratulations! Your manuscript is now with our production department. 

If your institution or institutions have a press office, please let them know about your upcoming paper now to help maximize its impact. If they'll be preparing press materials, please inform our press team within the next 48 hours. Your manuscript will remain under strict press embargo until 2 pm Eastern Time on the date of publication. For more information please contact onepress@plos.org.

If we can help with anything else, please email us at plosone@plos.org. 

Thank you for submitting your work to PLOS ONE and supporting open access. 

Kind regards, 

PLOS ONE Editorial Office Staff

on behalf of

Dr. Vanessa Carels 

Staff Editor

PLOS ONE
